# Surgical Treatment of an Immature Short-Rooted Traumatized Incisor with an Extensive Apical Lesion Using CEM Cement

**Published:** 2015-03-18

**Authors:** Saeed Asgary, Mahta Fazlyab

**Affiliations:** a * Iranian Center for Endodontic Research, Research Institute of Dental sciences, Dental School, Shahid Beheshti University of Medical Sciences, Tehran, Iran*

**Keywords:** Biomaterials, Calcium-Enriched Mixture, CEM cement, Endodontic, Periapical Cyst, Periradicular Lesion, Root-End Surgery

## Abstract

Severe traumatic injuries to immature teeth often cause damage to periodontal ligament as well as dental pulp; pulp necrosis, root resorption and subsequent apical lesion are common consequences. This article reports the surgical management of an infected immature maxillary central incisor associated with a gigantic periradicular lesion and severe root resorption. The tooth had a history of trauma and the patient suffered from purulent sinus tract and tooth mobility. After unsuccessful multi-session disinfection with calcium hydroxide, root end surgery was planned. During flap surgery and lesion enucleation, the root end was cleaned and filled with calcium-enriched mixture (CEM) cement. After one year, the radiographic examination revealed that the lesion was almost completely replaced with newly formed bone. In addition, clinical examination showed favorable outcomes; the tooth was symptom-free and in function. Due to chemical, physical and biological properties of CEM cement, this biomaterial might be considered as the root-end filling material of choice.

## Introduction

The general objective of endodontic treatment is to retain the treated tooth in normal function, and the specific goal is to prevent/cure apical periodontitis (AP) [1]. AP is defined as the inflammation of periodontium at the root canal connection portals [2]. It stems from the infectious root canal system, surrounding dentin and in some cases from the tissues outside the apical foramen. Thus the lesion is typically located at the root apex, but it may exist anywhere along the root near the lateral and furcal communication portals [2]. AP shows the classical features of inflammation. AP develops as a response to infection, and in the chronic phase a granuloma is formed with characteristics peculiar to the location and anatomy. However, if the cemental layer is lost or damaged (especially after trauma), the inflammatory stimulators will result in both bone and root resorption [3]. The clinical symptoms of acute AP include pain, swelling and sometimes increased temperature and impaired function. 

Traumatic dental injuries (TDI) are among the causes of AP [4]. Two main scenarios are involved in the development of pulp necrosis following TDI that can occur separately or simultaneously: coronal or apical invasion of bacteria through dentinal tubules and via exposed pulp or severance of the neurovascular supply with subsequent bacterial infection of the ischemic pulp so that revascularization of the pulp cannot occur [5]. In limited cases, the asymptomatic pulp necrosis ends up in large AP lesions many years later [4, 6].

Calcium hydroxide (CH) has been used as the intracanal medicament of choice to eliminate pathogenic intracanal bacteria due to its high pH level [7]. The hydroxyl ions in CH diffuse into the dentinal tubules and accessory canals, which induce an alkalizing effect and destroy the bacterial cellular membranes/protein structures. In addition, it has the ability to promote an osteogenic environment and prevent root resorption [8]. Some authors believe that multiple-session endodontic treatment of teeth with AP with inter appointment CH therapy can help in elimination of large AP lesions [9]. 

**Figure 1:**
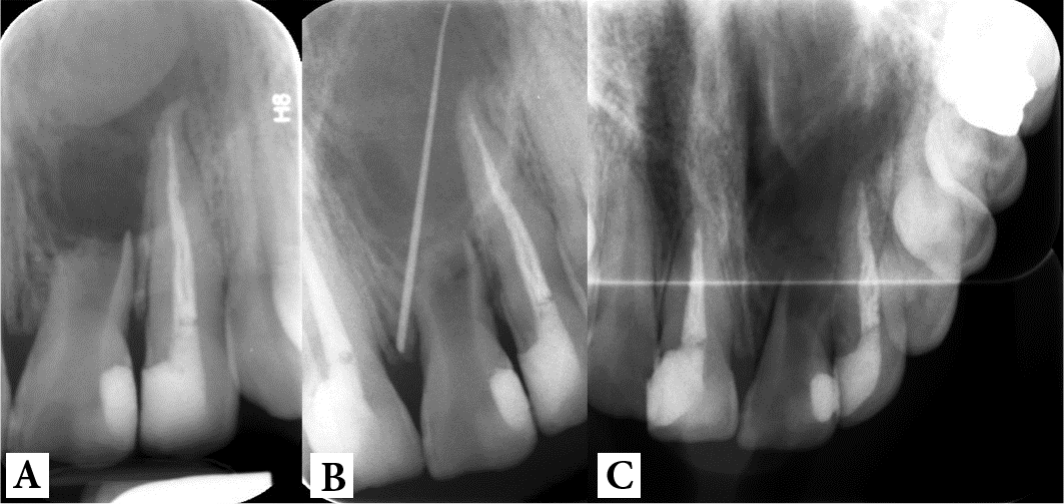
Preoperative radiographs: *A) *periapical;* B) * tracing with gutta-percha and* C) *occlusal view

The differentiation of cysts and granulomas is not possible by traditional radiographic techniques [10]. It is suggested that cyst cavities may have lower densities than granulomas, in computer tomography [11]. Several radiographic features have been proposed, including large lesion size and the presence of a radiopaque rim demarcating the cystic lesion [12]. 

The main aim of surgical endodontics is to achieve an apical seal [13]. Many properties are named for an ideal root-end filling biomaterial such as providing a good apical seal, adherence to the canal walls, dimensional stability, easy handling, radiopacity, nontoxicity and noncarcinogenicity, antimicrobial activity and cementogenic activity [13, 14]. As a root-end filling biomaterial, calcium-enriched mixture (CEM) cement has shown unique properties regarding film thickness and flow rate [15], antimicrobial activity [16], hydroxyapatite formation over the resected root-end and root-end filling [14, 15] and PDL regeneration [14]. All these properties put CEM cement in the list of ideal materials for root-end sealing.

The present case represents the trial and error treatment of a traumatized immature upper central incisor with root resorption and the least acceptable root length that was surrounded with an excessively large AP; treatment protocol started from orthograde endodontic intervention and ended up in periradicular surgery using CEM cement. 

## Case Report

A young man in his early twenties came to Mehr dental clinic complaining of a dull ache sensation in the anterior segment of the maxilla and mobility of the maxillary left central incisor. His medical history was not contributory and he reported an impact trauma at primary school. On clinical inspection, the tooth had a grayish hue and had grade II mobility. A draining sinus tract was evident 3 mm far from the gingival margin. The primary periapical radiography showed a very extensive periapical lesion surrounding the short/resorbed root of the tooth. In addition, the large size of pulp canal space was suggestive of tooth becoming necrotic before its maturation, which seemed accordant with the time of trauma. The distal margin of the lesion could not be defined after inserting the full length of a #30 gutta-percha cone into the sinus tract. An occlusal radiography confirmed the extremely large diameter of the lesion (Figure 1).

**Figure 2:**
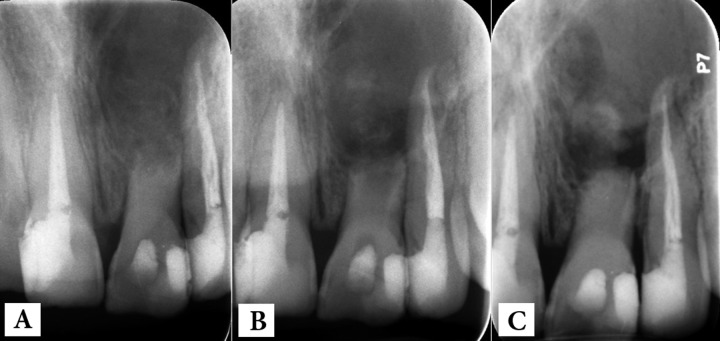
Post treatment radiographs: *A) *immediately after CH therapy;* B) *one week later (CH washing out) and* C) *CH replacement

After informing the patient of the hopeless condition of the tooth and possible treatment plans including saving the tooth via endodontic intervention with questionable prognosis, he insisted on giving a chance to the tooth and signed an informed consent.

Subsequent to tooth isolation and preparation of a classic access cavity; the pus and exudate flew through the access. After almost 15 min, the exudate ceased and canal was constantly irrigated with sterile normal saline and then 5% NaOCl that was kept in the canal for 5 min. Then the irrigation solution was dried and the canal was filled with thick paste of calcium hydroxide (CH) (Golchay, Tehran, Iran) mixed with saline; the access cavity was temporarily sealed. After taking the postoperative radiography, the patient was dismissed. One week later, the intracanal medicament had washed away (Figure 2) and the sinus tract was still present. The canal was reopened and refilled with CH and the tooth was temporarily restored with light-cured glass ionomer (Fuji II LC, GC Corporation, Tokyo, Japan). Two weeks later the patient came up with purulent sinus tract; the tooth was still mobile and periapical radiography revealed the presence of the intracanal medicament in canal as well as the lesion (Figure 2). 

The situation was discussed with the patient and he insisted on maintaining the tooth as long as possible. Endodontic surgery, curettage of the lesion and CEM root-end was offered as the final chance of saving the tooth. In the same session after disinfection with 0.2% chlorhexidine mouth rinse (Shahrdaru, Tehran, Iran) and profound local anesthesia, a full rectangular mucoperiosteal flap was raised. As it could be foretold, the buccal bone plate was destroyed but the osseous crest was still present. After enlarging the fenestration with engine-driven saline-cooled round drill, the underneath large cavity was curetted and cleaned. Multiple tissue segments were kept in 10% formalin for histopathological evaluation. The cavity was cleaned to the extent that the iatrogenic perforation of the nasal floor as well as maxillary sinus did not occur. Then the root canal was retrogradely cleaned with an ultrasonic device (Joya Electronic, Tehran, Iran). Then the canal was dried with sterile cotton pellets. CEM cement (BioniqueDent, Tehran, Iran) was prepared according to the manufacturer’s instructions. The biomaterial was carefully inserted into the canal with a plastic instrument and incrementally condensed with a tiny condenser.

**Figure 3:**
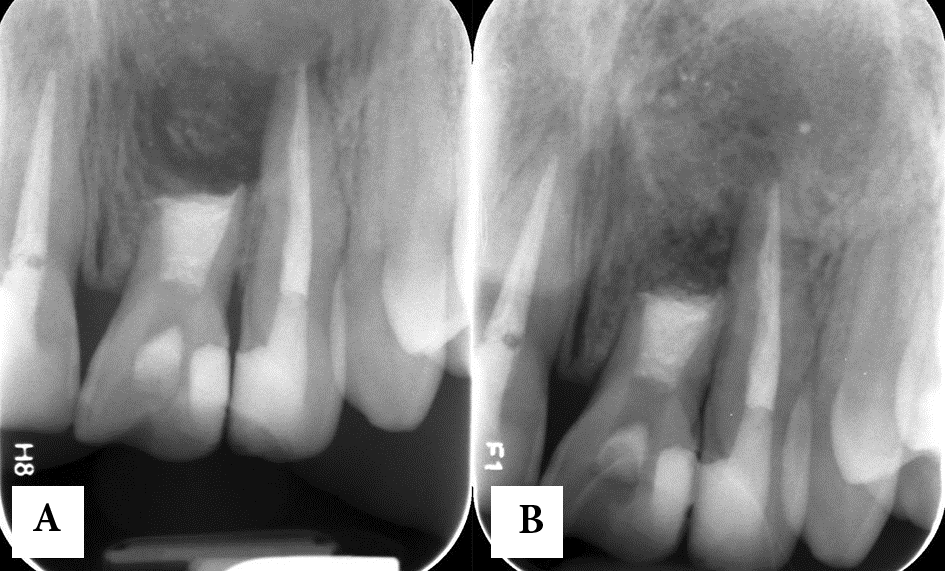
follow-up radiographs: *A) *immediately after surgery and* B) *One year later

After filling of the canal with CEM cement and taking a confirmation radiography (Figure 3), the light creamy mixture of CEM cement was placed in the cystic cavity and gently mixed with patient’s blood. The flap was replaced and sutured. Systemic oral antibiotic (500 mg amoxicillin and 250 mg metronidazole q8h for 7 days) and daily mouth rinsing with 0.2% chlorhexidine was prescribed. The patient was dismissed and one week later, the sutures were removed. He did not mention any discomfort after surgery and the sinus tract had disappeared. 

He did not attend the appointments set for final restoration of the access cavity and follow-up. One year later the patient accidentally appeared in the clinic and follow-up radiography was taken (Figure 3). Surprisingly, despite of not receiving any permanent restoration, the gigantic pretreatment periradicular lesion was almost totally replaced with new bone; in spite of less than 1:1 crown/root ratio, the tooth was totally asymptomatic and functional. During the same session the access cavity was permanently restored with composite resin.

## Discussion

This report represented the combined ortho/retrograde intervention for an infected/traumatized and extremely root resorbed upper central incisor associated with a very large periradicular lesion. The retrograde canal filling was done with CEM biomaterial and after one year, although the tooth had extremely short root length, it was completely functional/asymptomatic and the lesion had healed with newly formed bone. 

We did not employ triple antibiotic, because of discoloration potential of minocycline. Currently, placement of CH as intracanal medicament, for achieving successful outcome, is a controversial issue [17]; however, in the present case, CH therapy was unable to eliminate infection. 

In some cases, endodontic treatment is weighed against tooth extraction/replacement and being able to predict the treatment outcome is the key to clinical decision making [18]. Technical advances and the development of new biomaterials promise greater efficiency and improved endodontic treatment outcomes [19]. However, there is an air of concern as viable teeth, which could be treated/retreated endodontically, are being extracted in favor of dental implants [20]. Some of the reasons for such treatment option include the unfavorable crown to root ratio, insufficient root length, questionable periodontal status, and the condition of the surrounding dentition [21]. Considering the least acceptable crown/root ratio and financial conditions of our patient, surgical intervention (for this case,*herodontics*) was chosen instead of tooth extraction/implant placement. Implant treatment planning may involve separate examinations by the surgeon and restorative dentist and a variety of imaging techniques. A cost-benefit comparison between endodontic treatment and a single-tooth implant shows that the former is less expensive, entails fewer office visits and functionalizes the tooth more quickly than the implant [22]. Cost and time have been recognized as barriers to public acceptance of implants. Our patient insisted on maintaining the tooth as long as possible, keeping implant as a valuable option at all times.

Prevention/curing the AP is the top goal of endodontics which is not always achieved [1, 2]. In many circumstances, surgical endodontics becomes a necessary step in eradication of AP [23]. After surgery, healing begins with the regeneration of the external cortical plate and proceeds from the outside of the lesion toward the inside; scar tissue can also develop [24]. Historically, the literature has been inconsistent concerning the preoperative size of the lesion and surgical healing. There is no clear consensus that small lesions (<5 mm) heal more favorably than larger lesions. Lesions >10 mm show a lower rate of healing and a greater incidence of healing by scar tissue [25]. The gigantic AP in the present case, however, favorably healed after endodontic surgery.

There is a long tradition of long-term follow-up assessment of treatment outcome in endodontics. As a dynamic process, healing of AP requires sufficient time to evaluate its progression and completion [1]. Short follow-up observations may indicate only signs of healing [26]. At least one-year follow-up is required to reveal meaningful changes, but extension of the follow-up period to 3 or 4 years may be required to record a stable treatment outcome, as a Toronto study revealed healing of 86% of the treated teeth 4-6 years post-treatment [1]. During long time, endodontically treated teeth are subject to adverse effects of periodontal and restorative deterioration, thus longer follow-up periods are more likely to reveal the influence of those effects on the outcome [25]. Here, our patient neglected the post-surgery visits and within the one-year delay, the tooth was serendipitously kept sealed with intra canal CEM cement.

The last but not the least is the quality of apical seal provided by CEM biomaterial. Slight expansion, reasonable flow/film thickness, good adaptation as well as precipitation of hydroxyapatite over its surface, can ensure an effective seal. In addition, the high alkalinity of CEM cement provides a bactericidal environment that prepares the situations required for normal bone healing [15]. 

## Conclusion

The present case confirms the profound abilities of CEM cement to induce healing with normal bone formation in seemingly impossible/hopeless cases with a combined ortho/retrograde approach. 
